# Differential Lipid Composition and Gene Expression in the Semi-Russeted “Cox Orange Pippin” Apple Variety

**DOI:** 10.3389/fpls.2017.01656

**Published:** 2017-09-26

**Authors:** Sylvain Legay, Emmanuelle Cocco, Christelle M. André, Cédric Guignard, Jean-Francois Hausman, Gea Guerriero

**Affiliations:** ^1^Luxembourg Institute of Science and Technology, Belvaux, Luxembourg; ^2^Institut des Sciences de la Vie, Université Catholique de Louvain, Louvain-la-Neuve, Belgium

**Keywords:** apple, russeting, suberin, cutin, triterpenes, waxes, lipid composition

## Abstract

Russeting is characterized by a particular rough and brown phenotype, which is mainly due to the accumulation of suberin in the inner part of the epidermal cell walls. In our previous bulk transcriptomic analysis, comparing fully russeted, and waxy apple varieties, showed, in apple fruit skin, a massive decreased expression of cutin, wax and some pentacyclic triterpene biosynthesis genes in the russeted varieties, with an expected concomitant enhanced expression of the suberin biosynthetic genes. In the present work, we performed a deep investigation of the aliphatic composition of the cutin, suberin, waxes, and triterpenes in the waxy and russeted patches of the semi-russeted apple variety “Cox Orange Pippin.” A targeted gene expression profiling was performed to validate candidate genes which were identified in our previous work and might be involved in the respective metabolic pathways. Our results showed that a decrease of cuticular waxes, ursolic acid and oleanolic acid, accompanied by an accumulation of alkyl-hydroxycinamates and betulinic acid, occurs in the russeted patches. The suberin monomer composition is characterized by specific occurrence of 20, 22, and 24 carbon aliphatic chains, whereas cutin is mainly represented by common C16 and C18 aliphatic chains. This work depicts, for the first time in apple, the complex composition of suberin, cutin, waxes and triterpenes, and confirms the strong interplay between these epidermal polymers in apple fruit skin.

## Introduction

Apple is among the most consumed fruits worldwide with approximately 81 million tons produced in 2013 (FAOSTAT). The *Malus* × *domestica* species have a long history of breeding and cross hybridization which started by the import of the first *Malus sieversii* cultivars in Europe, through the Silk Route (Cornille et al., 2012, 2014). Later, breeding efforts aimed at improving yield, tolerance to pathogens and fruit quality traits, such as flesh firmness, juiciness, sugar, and polyphenol contents, to name a few (Laurens, 1998; Morgan et al., 2002; Juniper and Mabberley, 2006). Since the last decades, consumers and breeders have been progressively more attentive to the skin phenotype, favoring the development of glossy and colored skin varieties also called “waxy” varieties. However, the production of fruits with no damage is extremely dependent on environmental pressures and compels farmers to use increasing quantities of pesticides and fertilizers (Faust and Shear, 1972). Despite their efforts, some defects on the fruit surface can still occur. Among these, russeting is one of the most important causes of economic loss in the apple production sector. Fruits exhibiting this rough brown phenotype on the surface are usually redirected to the transformed product sector with a concomitant price downgrading, compared to the consumer's market.

The common apple skin surface is constituted by a thick cuticle linked to the epidermal cell walls of external tissues acting as protective barrier against environment. The cuticle composition varies across plant species and its chemical composition is characterized by the presence of multiple classes of hydrophobic hydrocarbons. Indeed, the cuticle is composed of a covalently linked macromolecular scaffold of cutin constituted by C16 and C18 ω-hydroxyacids and α,ω-dicarboxylic acids, and a wide variety of organic solvent-soluble lipids including n-alkanes or primary alcohols to name a few (Yeats and Rose, 2013; Fernández et al., 2016). The cuticle structure has been divided in multiple domains composed by (i) an internal cuticular layer linked to the cell wall, which is rich in cutin polymer with embedded polysaccharides, such as pectins and cellulose, (ii) an external cuticle (cuticle proper) with lower amount of polysaccharides but enriched in embedded intracuticular waxes and finally (iii) the epiculticular waxes which accumulate in the form of crystals or films responsible for the glossy phenotype of the apple fruit skin (Yeats and Rose, 2013; Fernández et al., 2016). Notably, a second non-saponifiable polymer called cutan has been observed in some species including apple (in low extent, Johnson et al., 2007). Cutan is rich in ether and carbon-carbon bounds but its structure remains poorly understood until now (Yeats and Rose, 2013; Fernández et al., 2016). Russeting is mainly due to the accumulation of suberin in the inner part of the primary cell wall. This complex biopolymer has been widely studied in other plant models, such as *Arabidopsis thaliana, Solanum tuberosum* L., and *Quercus suber* L. (Graça and Pereira, 2000a,b; Franke et al., 2005; Molina et al., 2006). Although recent advances strongly improved the knowledge of the complex metabolic cascade resulting in the deposition of suberin in the primary cell wall, factors driving its biosynthesis remain still uncharacterized in apple and, more generally, in plants (Graça, 2015; Legay et al., 2015; Vishwanath et al., 2015). Since the 60's, russeting has been extensively studied at the agronomical and histological levels, and key factors favoring the development of russeted skin in apple have been identified (Sironval and Clijsters, 1962; Walter, 1966; Tukey, 1969; Faust and Shear, 1972; Skene, 1982; Knoche and Grimm, 2008; Lashbrooke et al., 2015). Among these, the development and the integrity of the cuticle seems to be one of the most critical points in the regulation of russeting (Lashbrooke et al., 2015). Indeed, the development a thinner cuticle is correlated with the occurrence of skin russeting. This altered cuticle appears to be linked with the appearance of cuticle microcracking on the apple fruit surface and is thought to be one of the major causes of russeting in apple (Knoche et al., 2011; Curry, 2012). The cuticle is particularly sensitive to microcracking during early stages of fruit development, due to the extreme tangential tensions on the surface generated by the expansive growth (Khanal et al., 2012). Moreover, rheological studies showed that the skin of the non-russeted apple is less elastic compared to the russeted periderm, suggesting that the cuticle might be more sensitive to microcracking compared to the suberized tissue (Khanal et al., 2012). Scanning electron microscopy (SEM) analyses performed on the “Golden Delicious” variety showed that gibberellins (GA_4+7_) application during the early stage of fruit development decreases the epidermal cell surface and fruit surface tensions, leading to a lower cuticle microcracking rate and russeting (Curry, 2012). In addition to the developmental factors, environmental factors strongly impact russeting in apple. As an example, the long term moisture exposure has been also associated with cuticle microcracking and russeting, suggesting that the causes leading to russeting are multifactorial (Tukey, 1969).

In our previous bulk transcriptomic analysis comparing russeted and waxy apples, a large number of genes involved in the cutin, wax and suberin biosynthesis pathway have been identified and new putative candidate genes implicated in these metabolic processes have been highlighted too (Legay et al., 2015). Interestingly, a strong decreased expression of the cutin and wax biosynthetic genes, with a concomitant increased expression of suberin biosynthetic and cell modification transcripts, was observed in russeted skin. Cutin and suberin share a similar backbone composed of α,ω-dicarboxylic acids and ω-hydroxyacids esterified with glycerol (Bernards, 2002). However, strong differences occur between these biopolymers, which might explain their differential rheological properties observed in apple (Khanal et al., 2012). As an example, cutin displays higher relative content in ω-hydroxyacids, which are composed by a majority of C16 and C18 aliphatic chains. Conversely, suberin is constituted by distinct polyphenolic and polyaliphatic domains, which contains significant amount of hydroxycinnamic acids, mainly ferulic acid, which are esterified within the suberin structure (Graça and Pereira, 2000b; Bernards, 2002). The suberin polyaliphatic domain also displays characteristic very long chains ω-hydroxyacids and α,ω-dicarboxylic acids, with a higher relative content in these latter compounds (Graça and Pereira, 2000a,b; Franke et al., 2005; Molina et al., 2006). Suberin composition displays variable distribution of these suberin precursors among plant species (Graça and Pereira, 2000a; Franke et al., 2005; Ranathunge and Schreiber, 2011; Vishwanath et al., 2015). In *Q. suber*, C22, and C24 ω-hydroxyacids and α,ω-dicarboxylic acids are predominant, while a majority of C16-C18 chains is observed in *Pseudotsuga menziesii* (Graça and Pereira, 2000a). In the potato tuber periderm, in addition to the long chain alkanoic acids (C24-C28), a majority of 18:1 derived ω-hydroxyacids and α,ω-dicarboxylic acids is found (Graça and Pereira, 2000b). Moreover, a variable composition of suberin is also observed between tissues of the same species. As an example, a differential suberin composition has been observed between *Arabidopsis* root and seed coat. Root suberin displays a large majority of C16, C18:1, and C22 α,ω-dicarboxylic acids, whereas seed coat suberin shows longer chains (Franke et al., 2005; Molina et al., 2006). In plants, cutin and suberin are complexed with non-covalently linked waxes which differ in composition between these two types of polymers (Millar et al., 1999; von Wettstein-Knowles, 2001; Schreiber et al., 2005; Rowland et al., 2007; Kosma et al., 2012). Waxes found in the cuticle are composed by very long chain fatty acids, primary and secondary alcohols, aldehydes and ketones, alkanes, wax esters and alicyclic components, such as triterpenes, which are deposited on the cutin surface (epicuticular waxes), or embedded within the polymer matrix (intraculticular waxes) (Aarts et al., 1995; Rowland et al., 2007; Lü et al., 2009; Zeisler and Schreiber, 2015; Jetter and Riederer, 2016). Recent studies showed that the transpiration barrier property of the cuticle is highly dependent to the fatty acyl content and to their intracuticular localization (Zeisler and Schreiber, 2015; Jetter and Riederer, 2016). Conversely, the suberin-associated waxes comprise very long chain alkanes, primary alcohols, fatty acids, some sterol derivatives and alkyl-hydroxycinnamates. Alkyl-hydroxycinnamates, which are composed by hydroxycinnamic acid esterified with primary alcohols, can reach 60% of the total waxes weight in *A. thaliana* (Kosma et al., 2012). Moreover, in *A. thaliana*, a recent study showed that the large majority of the primary alcohols is non-covalently linked to suberin is in the form of alkyl-hydroxycinnamates (Delude et al., 2016).

The apple waxy and russeted (suberized) skins present some important specificities in their wax composition compared to *A. thaliana*. Indeed, in the waxy apples, massive contents of the pentacyclic triterpenes, ursolic and oleanolic acids are found and can reach 30–60% of the total content depending on the variety (André et al., 2012, 2013). Conversely, russeted skins contain a lower amount of these compounds, but accumulate betulinic acid and lupeol (André et al., 2012, 2013). Moreover, betulinic acid-3-*trans*-caffeate also specifically accumulates in this suberized tissue (André et al., 2013). This suggests that, in apple, there might be a close interplay between the suberin, cutin and triterpene biosynthesis pathways, although no evidence has been established so far.

The purpose of this study is to characterize for the first time, in apple, the differential distribution of the cutin, suberin, waxes, and triterpenes, which occur between waxy (normal) and russeted (suberized) tissues. For this, a deep investigation of the lipid surface composition was performed from normal waxy skin and russeted patches sampled from the semi-russeted fruits of the “Cox Orange Pippin” variety. “Cox Orange Pippin” is a famous and well-studied apple variety, which has been used as parent in a large number of available commercial varieties (Sironval and Clijsters, 1962; Knee, 1972; Skene, 1981, 1982; Irving and Drost, 1987; Evans et al., 2011; Chagné et al., 2012; Legay et al., 2015). The comparison of the russeted and waxy areas of the same variety provides a first picture of the differential accumulation of cutin, suberin, waxes and triterpenes, discarding genotypic factors that might occur when such a comparison is performed on different russeted/waxy varieties. The composition of these polymers and free lipid compounds was also compared with previous works performed in suberin model plants, such as *A. thaliana, S. tuberosum* and *Q. suber* (Graça and Pereira, 2000a,b; Franke et al., 2005; Molina et al., 2006). Finally, a targeted gene expression profiling of biosynthetic genes previously identified in apple has been performed using RT-qPCR. These results will be merged in order to decipher the key players involved in the differentiation of these two contrasting tissue types.

## Materials and methods

### Plant material

For the lipid composition and the qPCR analysis, four trees of the “Cox Orange Pippin” variety (four biological replicates) were randomly sampled at the commercial harvest stage (120 days after full bloom) from the orchard of the Walloon Agronomic Research Center of Gembloux (CRA-W) in 2014. From each tree, 10 apples were sampled from the south side of the tree at a height ranging from 1.20 to 2.20 m and represent a biological replicate. For each biological replicate, the russeted and waxy patches (areas) were carefully excised with scalpels taking care to remove the flesh as much as possible. The resulting exocarp samples were directly flash-frozen in liquid nitrogen and stored at −80°C until RNA extraction.

### RNA extraction

Samples (250 mg) were ground to a fine powder with a mortar and pestle in liquid nitrogen. Total RNA was extracted using an adapted CTAB buffer extraction protocol (Gasic et al., 2004). Total RNAs were cleaned and treated with DNase I using the RNeasy Plant Mini Kit (QIAGEN, Leusden, The Netherlands), following the manufacturer's guidelines. Total RNA integrity was assessed using the RNA Nano 6000 assay and a 2100 Bioanalyzer with quality parameters adapted to plant RNA profiles (Agilent Technologies, Santa Clara, CA, USA). Samples with RINs (RNA integrity numbers) lower than 7 were excluded from the experiment. RNA purity was assessed measuring the absorbance at 230, 260, and 280 nm using a Nanodrop ND1000 spectrophotometer (Thermo Fisher Scientific, Waltham, MA, USA). Total RNA was quantified using a Qubit RNA assay kit (Life technologies, Carlsbad, CA, USA).

### Targeted gene expression analysis

The targeted gene expression analysis was performed on 28 representative genes involved in cutin, suberin, wax, and triterpene biosynthesis, using RT-qPCR (Table 1). Reverse transcription was carried out using the M-MuLV Reverse Transcriptase (RNase H), the Murine RNase Inhibitor (New England Biolabs, Ipswich, MA, USA) and random hexamers (Invitrogen, Carlsbad, NM, USA) following the manufacturers' guidelines. qPCR primers were designed using the Primer3 software (http://frodo.wi.mit.edu/) with the following criteria: primer size between 18 and 25 base pairs, GC content between 30 and 70%, amplicon size from 80 to 200 base pairs, primer annealing temperatures in the 57–61°C range. Matching primer sets were checked for unexpected secondary structures using NetPrimer (http://www.premierbiosoft.com/netprimer). In order to test the specificity of the primers, a BLAST search against the *M*. × *domestica* genome sequence was performed (www.rosaceae.org). qPCR runs were carried out using technical triplicates and four biological replicates on a Viia7 384-well real-time PCR instrument (Thermo Fisher, Waltham, MA, USA) with Takyon SYBR Green low ROX (Eurogentec, Liege, Belgium) following the manufacturer's guidelines. Experiments were carried out following the MIQE guidelines (Bustin et al., 2009) and a melting curve was performed at the end of all the runs to assess the specificity of the primers. Primer information is available in Table 1 and Supplementary Material Table 1. The relative expression of a gene of interest was calculated using the Biogazelle qBase+ V3 software (Hellemans et al., 2007), taking into account multiple reference gene normalization and specific PCR efficiencies. For the present experiment, the geNorm+ analysis described the *MdIMPA9* and *MdActin* gene couple as the most suitable for optimal normalization of the data (Vandesompele et al., 2002; Giorno et al., 2012). A statistical analysis of the log_2_ transformed normalized relative quantities was performed between both groups (russeted and waxy skins) using a Mann-Whitney test in Biogazelle qBase+ V3.

**Table 1:** Description of the 28 representative genes involved in the cutin, suberin, wax and triterpene biosynthesis.

	**Description**	**Targeted contig/EST**	**Primer sequence (5′-3′)**		**Amplicon size (bp)**	**PCR efficiency**	**References**
ASFT	Arabidopsis suberin feruloyl transferase	MDP0000312405	F:	GAGCCACCAGTACCAGAAGG	105	2.07	
			R:	ACCCAACTGCAAATGAAAGC			
FAR5	Fatty acid reductase 5	MDP0000138841	F:	CCGGGAGACGTAGTTTCTGA	105	2.00	
			R:	GTTGGTGGTTCCTGCAGAGT			
KSC4	3-ketaoacyl-CoA Synthetase 4	MDP0000922301	F:	GAACTATGTTCCGCCAAGGA	83	1.87	
			R:	ACAGATCATCCAATGCACCA			
CER1	Eceriferum 1	MDP0000461409	F:	TTCCAAATTGGCTCTTCACC	81	1.99	
			R:	GTGTGTGGTGCAAAGAATGG			
KCS2	3-ketaoacyl-CoA Synthetase 2	MDP0000625646	F:	GGTGCAGACGACAGAGCATA	134	1.93	
			R:	GGGCCAAGAGTTGTGATGTT			
CER3	Eceriferum 3	MDP0000165547	F:	CCAATGTTGAGGTCGTTCCT	117	1.88	
			R:	GGCAGAAGTTGGTGTCCATT			
CER6	3-ketaoacyl-CoA Synthetase 6/Eceriferum 6	MDP0000392495	F:	CTTCAAACCTCCGGTCATGT	99	1.88	
			R:	GACGCTCTTAGGGTTGTTGC			
C4H	Coumarate 4-hydroxylase	MDP0000602841	F:	GGCTAATGGGAACGACTTCA	85	1.97	
			R:	CATGATTGGAAGGGCTAGGA			
CER8/LACS1	Long chain acyl-CoA synthetase 1	MDP0000465035	F:	CCGCGTGATTGAGGAGTATT	88	2.02	
			R:	CACCATGTCATCCCTCAGTG			
CYP86A2	Fatty acid hydroxylase	MDP0000858983	F:	TTCCAAATGGGTGAAAGAGC	104	2.00	
			R:	GTGGCACAGAAGGGTAAAGC			
LCR/CYP86A8	LACERATA/Fatty acid hydroxylase	MDP0000941955	F:	AGCTTGAACCATAGCCTCCA	102	1.97	
			R:	CCCATTGCAAAGACCTTGTT			
MYB52	MYB domain transcription factor 52	MDP0000852158	F:	TTGTAGCTGCAACCCTCCAC	122	2.01	
			R:	ACCACCAACACCTATGAATGCT			
KCS10	3-ketaoacyl-CoA Synthetase 10	MDP0000235280	F:	GTGTACCAAGAGGAAGATGAACAAA	123	1.92	Legay et al., 2015
			R:	GAAAGGCAGCACTAACGGC			
CYP86B1	Very long chain fatty acid hydroxylase	MDP0000306273	F:	CGCTTTGTGACCCCATCC	114	1.98	Legay et al., 2015
			R:	AATGACGTCTTCCGCAAACT			
GPAT6	Glycerol-3-phosphate acyltransferase 6	MDP0000479163	F:	CCCAATAGTTGCCCTTCTGA	154	1.98	Legay et al., 2015
			R:	AGGAGGGGTGCCCTTAACTA			
CYP86A1	Fatty acid hydroxylase	MDP0000923760	F:	GCGCCTCCTCTATCCTTCC	89	1.94	Legay et al., 2015
			R:	CGACTTCGAGGCTCTTCTCC			
MYB42	MYB domain transcription factor 42	MDP0000787808	F:	GACCCGTTGGTGAGTTTCAT	104	2.00	Legay et al., 2015
			R:	TCCTCCGTTGGATTATCAGC			
WSD-1	O-acyltransferase	MDP0000701887	F:	AGAAATGGTCAAACCCGACA	107	1.91	Legay et al., 2015
			R:	AGACGAAGTCAAGCGCATTT			
MYB93	MYB domain transcription factor 93	MDP0000320772	F:	TATCCAACACCACCAAGCAA	145	1.96	Legay et al., 2015
			R:	CCACCGAAGTAGGAGATGGA			
GPAT5	Glycerol-3-phosphate acyltransferase 5	MDP0000150502	F:	GAACAAATCCACCCACCACT	131	1.97	Legay et al., 2015
			R:	ATTAAGAGGGCGGTTGAAGG			
MdOSC1	Oxidosqualene cyclase 1	FJ032006.1	F:	TTGTACTACTAATCCAGTGATCAAGATGTGG	238	1.86	Brendolise et al., 2011
			R:	CTCTCTTAGTATCTGAAAACGCCATAGGAG			
MdOSC2	Oxidosqualene cyclase 2	FJ032007.1	F:	CGCAGATGGTGGCAATGATCCATACATC	206	1.86	Brendolise et al., 2011
			R:	TGAAGTTCTTCTCCCTTAAGAACTGCATTC			
MdOSC3	Oxidosqualene cyclase 3	FJ032008.1	F:	GCAATCGTGATCAAAGAAGATGTGGAGG	232	1.82	Brendolise et al., 2011
			R:	TTCTCTTAAAATCTGAAAACGCCATAGG			
MdOSC4	Oxidosqualene cyclase 4	MDP0000034165	F:	TATGCATCCAGCAAAAATGTTT	124	1.94	André et al., 2016
			R:	TCCAATTAATTTCGCCATAAGGT			
MdOSC5	Oxidosqualene cyclase 5	KT383436	F:	CTCTCGAAGTAACAATGAAGCACA	152	1.97	André et al., 2016
			R:	TAATCACCATTTGGGTCTTCAG			
CYP716A145	Cytochrome P450 monooxygenase	MDP0000478473	F:	AGGCACGTTCCTCGCTTC	70	2.04	André et al., 2016
			R:	CAAACCCTAAGAGGAGGGTCA			
SQS	Squalene synthase	CN880837.1	F:	GTTTTGGCCACGTCAAATCT	87	2.12	André et al., 2016
			R:	CATTGCACTGCCTTCTCTGA			
eF-1alpha	Elongation factor 1 alpha	AJ223969.1	F:	ACTGTTCCTGTTGGACGTGTTG	208	1.95	Giorno et al., 2012
			R:	TGGAGTTGGAAGCAACGTACCC			
ACTIN	Actin	EB127077	F:	ACCATCTGCAACTCATCCGAACCT	185	1.94	Giorno et al., 2012
			R:	ACAATGCTAGGGAACACGGCTCTT			
IMPA-9	imortin subunit alpha-9	CN909679	F:	TCGTGAACTCAGGCGCTTACTG	205	1.92	Giorno et al., 2012
			R:	AAGCAACGGTAAAGCGGGCAAC			
GAPDH	glyceraldehyde 3-phosphate dehydrogenase	EB146750	F:	TGAGGGCAAGCTGAAGGGTATCTT	185	1.91	Giorno et al., 2012
			R:	TCAAGTCAACCACACGGGTACTGT			

*Primer sequences were obtained from previous studies or designed from the differentially expressed genes observed in our previous transcriptomic data*.

### Cuticle isolation

For each biological replicate, the total apple peel surface was measured and transferred into a 50 mL polypropylene tube. Cell wall and flesh residues were removed using 12 mL of a solution of pectinase and cellulase (250 U/mL each, Novozymes, Bagsvaerd, Denmark) in a 0.1 M sodium citrate/critic acid buffer pH = 4.2 for 3 days on a rolling table at 60 Hz at room temperature. After 3 days, the solution was removed and the samples were washed using 12 mL sodium borate/boric acid buffer (0.01 M, pH = 9) and 12 mL of a fresh cellulose/pectinase digestion solution in sodium citrate/critic acid buffer was added. Seven consecutive digestion/washing cycles were performed. After a final wash with 12 mL sodium borate/boric acid buffer (0.01 M, pH = 9) and 12 mL mQ water, samples were dried in an oven at 35°C for 48 h and transferred into a 2 mL centrifuge tube. Samples were then ground using 2 steel beads (4.5 mm) and a Retsch MM400 cryogenic grinder at 25 Hz for 5 min (Retsch, Aartselaar, Belgium).

### Cutin and suberin analysis

To extract the soluble lipid fraction, three consecutive extractions were performed with 1 mL of dichloromethane by shaking at 25 Hz for 10 min. The extracts were then evaporated under vacuum (CentriVap, Labconco, Kansas City, MO, USA) and recovered in 1 mL of dichloromethane. The remaining material was then oven-dried for 48 h at 35°C. Suberin depolymerization was performed according to the protocol described by Franke et al. (2005). Six milliliter of boron trifluoride methanolic solution (10% BF_3_/MeOH, Sigma Aldrich) were added to the powder in a glass tube sealed with a Teflon cap. After 16 h at 70°C, the transesterification reaction was stopped using 10 mL aqueous NaHCO_3_ saturated solution. An internal standard, i.e., nonadecanoic acid (4 μg), was added to the solution. The methylesters were then extracted 3 times (3 mL dichloromethane, 25 Hz for 10 min), evaporated, and resuspended in 1 mL dichloromethane. A 200 μL aliquot of the soluble lipid and suberin depolymerization extract in dichloromethane was placed in a GC-MS vial and dried under a gentle nitrogen flow, then 50 μL of a Bis-Trimethylsilyl-trifluoroacetamide: Trimethylchlorosilane mixture (BSTFA:TMCS 99:1) was added. The reaction was carried out for 60 min at 60°C, then the derivatized extract was directly analyzed by Gas chromatography-mass spectrometry (GC-MS).

The identification and quantification of derivatized compounds in both extracts were carried out on an Agilent 7890B/5977A GC-MS coupled to a Gerstel Multipurpose Sampler (Santa Clara, CA, USA). The injection was performed at 280°C in splitless mode (1 μL). The carrier gas was He at a constant flow of 1.2 mL/min. The column was an Agilent HP5-MS (30 × 0.25 mm i.d. × 0.25 μm film thickness) and the oven temperature was programmed as follows: 80°C (2 min) to 210°C (8°C/min), then 280°C (3°C/min) and 320°C (15°C/min, 15 min isotherm). The MS transfer line was kept at 280°C. MS spectra were recorded in Full-Scan mode between 50 and 900 amu. The identification of compounds in the soluble lipid and polymerized polyester fractions and the determination of their relative peak areas were performed using the Agilent MassHunter Software suite. The identification of the different metabolites was performed via comparison of the mass spectra with data from the National Institute of Standards and Technology (NIST) database (http://www.nist.gov), the American Oil Chemists' Society (AOCS) lipid library (http://lipidlibrary.aocs.org) and fragmentation patterns described in the literature (Bento et al., 1998; Graça and Pereira, 2000b; Graça et al., 2002; Franke et al., 2005; Molina et al., 2006, 2009; Kosma et al., 2012). GC-MS fragment tables are available in the Supplementary Material Table 2. For each compound, the relative peak area was normalized the C19 internal standard and the skin surface. The statistical significance was assessed using a Mann-Whitney test comparing the four biological replicates of the waxy and the russeted skin, respectively. The total amount of free and polymerized lipids was evaluated from the sum of the compounds identified in the GC-MS analysis.

## Results

### Cox orange pippin skin phenotype

**Figure 1:**
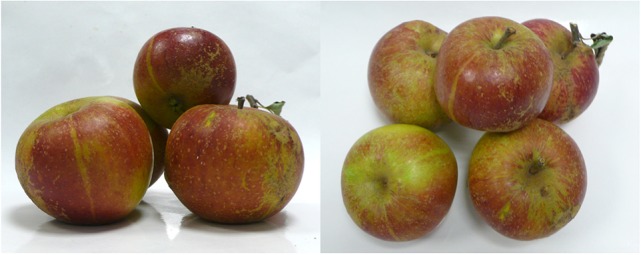
The “Cox Orange Pippin skin phenotype.” On the left: side view, on the right: top view. The majority of the fruit surface was covered by a waxy skin with small lenticels (russeting spots). Russeting was systematically found on the calyx and stalk ends of the fruit and russeted patches occurred aleatory on the fruit surface.

The “Cox Orange Pippin” variety harvested in 2014, in the orchards of the CRA-W, showed a quite high russeting coverage on the fruit surface (Figure 1). The calyx and stem ends were heavily russeted as observed in a majority of commercial apple varieties. Some russeted patches were also present on the remaining fruit surface with no evident relation with the fruit orientation in the tree. In addition, some characteristic russeted spots (lenticels) were observed on the whole surface of the fruit including the waxy areas. Sampling of the tissues was performed by carefully excising the russeted patches and the remaining waxy areas.

### Strong decrease of wax and triterpene contents in russeted tissues

Analysis of the free lipid fraction of the russeted and waxy skin of the “Cox Orange Pippin” variety showed a large diversity in metabolites composing waxes. Interestingly the total organic solvent soluble lipid concentration strongly differed between the waxy and russeted tissues, with 537.6 μg/cm^2^ (20.55% of the isolated cuticle) and 87.7 μg/cm^2^ (2.72% of isolated cuticle). This fraction was represented by common fatty acids, primary fatty alcohols, alkanes, aldehydes and triterpenes, which displayed drastic decreased concentrations in the russeted skin. Fatty acids exhibited a large diversity of chain length, which ranged from 16 carbons (C16) to thirty carbons (C30) (Table 2). The C16, C18, C18:1, and C18:2 fatty acid precursors, which are synthesized in the plastids, showed a higher accumulation in the waxy skin with fold changes equal to 2.6, 4.1, 3.2 and 4.1, respectively. The C26, C28 and C30 VLCFAs, which are commonly found in the waxes, strongly accumulated in the waxy skin with fold changes ranging from 9 to 17 compared to russeted skin. Interestingly, russeted patches showed a slightly higher accumulation of the C22 and C24 VLCFAs compared to the C26, C28, and C30, suggesting that these two VLCFAs groups might be synthesized by different 3-ketocyl-CoA synthases (KCS) (Millar and Kunst, 1997). Primary alcohols which comprise C18–C30 aliphatic chains, were found in both russeted and waxy skin. These also displayed a similar distribution compared to fatty acids, with an expected prevalence of even carbon chains (Table 2). In the waxy skins, the most abundant primary alcohols were composed by 26 and 28 carbons chains, which represented 34 and 35% of the total primary alcohol content. The C24 and C30 chains primary alcohols also accumulated, to a lower extent, in the waxy tissues and showed lower amount in russeted tissues as observed for the C26 and C28 primary alcohols. Other odd-carbon chain primary alcohols showed lower concentration in both tissues and a slight decreased accumulation in the russeted tissues. Finally, small amounts of octadecanol and eicosanol were detected and can be considered as trace. Only two aldehydes were found in the free lipid extract. Aldehydes were mostly represented by C26 and C28 chains, which accumulated 49 and 28 times more in the waxy skin, respectively. Interestingly, the carbon chain length of these aldehydes matched with the most abundant primary alcohols, supporting a link between these two families in the metabolic cascade of wax biosynthesis (See discussion). Alkanes were among the most abundant metabolites found in the free lipid fraction (Table 2). The C27 and C29 *n*-alkanes were the most represented forms with 19 and 75% of the total alkanes in the waxy skin, respectively, and 22% and 70% in the russeted skin, respectively. Although their relative abundance was similar between both skin types, the C27 and C29 *n*-alkanes accumulated 10 and 8 times more in the waxy skin, respectively, suggesting that the alkane biosynthesis was also impaired in russeted skins. Other alkanes, such as the even number chain length and the C31 accumulated to a much lower extent.

**Table 2:** Distribution of the identified fatty acids (as trimethylsilyl derivatives), fatty alcohols (as trimethylsilyl derivatives), fatty aldehydes, alkanes, triterpenes (as trimethylsilyl derivatives), alkyls-hydroxycinnamates (as trimethylsilyl derivatives) and miscellaneous metabolites found in the free lipid fraction of the waxy (non-russeted) and russeted skin.

	**Non-russeted skin**		**Russeted skin**		**Mann-whitney test, *p* < 0.05**
	**Mean (μg/cm^2^)**	**Standard dev**.	**Mean (μg/cm^2^)**	**Standard dev**.	
**CARBOXYLIC ACIDS**					
C16	4.65	0.89	1.77	0.61	Sig.
C18	5.11	0.75	1.26	0.41	Sig.
C18:1	2.24	0.57	0.68	0.21	Sig.
C18:2	0.46	0.13	0.08	0.02	Sig.
C20	2.68	0.43	0.56	0.15	Sig.
C20:1	0.30	0.07	0.04	0.01	Sig.
C22	3.30	0.75	1.14	0.32	Sig.
C24	5.22	0.73	1.28	0.42	Sig.
C26	4.35	0.82	0.48	0.10	Sig.
C28	5.78	1.24	0.38	0.07	Sig.
C30	5.59	1.72	0.32	0.06	Sig.
Total	39.67	8.11	7.99	2.39	
**PRIMARY ALCOHOLS**					
C18	0.29	0.04	0.08	0.02	Sig.
C20	0.17	0.02	0.06	0.01	Sig.
C22	1.75	0.49	0.43	0.55	
C24	5.87	0.45	2.22	0.88	Sig.
C25	0.93	0.23	0.13	0.03	Sig.
C26	23.03	3.44	2.91	0.75	Sig.
C27	1.53	0.28	0.15	0.02	Sig.
C28	23.34	4.54	2.15	0.51	Sig.
C29	1.69	0.29	0.19	0.02	Sig.
C30	7.44	1.74	1.39	0.26	Sig.
Total	66.05	11.51	9.72	3.05	
**FATTY ALDEHYDES**					
C26	1.62	0.35	0.03	0.00	Sig.
C28	7.11	1.68	0.25	0.06	Sig.
Total	8.74	2.03	0.29	0.06	
**ALKANES**					
C26	4.76	0.45	0.37	0.08	Sig.
C27	59.39	7.24	5.90	1.43	Sig.
C28	8.95	1.48	0.79	0.15	Sig.
C29	184.85	29.49	23.10	5.84	Sig.
C30	1.29	0.36	0.12	0.02	Sig.
C31	1.55	0.46	0.20	0.03	Sig.
Total	260.79	39.49	30.48	7.55	
**TRITERPENES**					
β-Amyrin	5.48	1.00	1.30	0.25	Sig.
Lupeol	1.13	0.45	2.73	1.08	Sig.
Oleanolic acid	20.78	3.80	1.93	0.49	Sig.
Ursolic acid	34.39	10.05	4.40	1.26	Sig.
Germanicol	0.62	0.12	0.44	0.19	
TTP1	2.26	0.46	0.20	0.02	Sig.
TTP2	1.99	0.85	1.88	0.73	
TTP3	1.80	0.51	0.47	0.15	Sig.
TTP4	4.94	1.66	2.10	0.93	Sig.
TTP5	2.44	1.56	0.99	0.40	
TTP6	0.21	0.37	0.16	0.10	
Total	76.04	20.81	16.60	5.59	
**ALKYLS-HYDROXYCINNAMATES**					
C22-coumarate	0.00	0.00	0.03	0.01	Sig.
C24-coumarate	0.00	0.00	0.12	0.06	Sig.
C26-coumarate	0.00	0.00	0.01	0.01	
C22-ferulate	0.00	0.00	0.01	0.01	Sig.
C24-ferulate	0.00	0.00	0.01	0.01	
C24-caffeate	0.00	0.00	0.02	0.01	Sig.
Total	0.00	0.00	0.20	0.10	
**MISC**.					
Glycerol	80.86	13.09	20.88	8.73	Sig.
C16-MAG	0.07	0.03	0.14	0.04	
C18:1-MAG	0.00	0.00	0.13	0.04	Sig.
β-sitosterol	5.48	1.00	1.30	0.22	Sig.

*Quantities are expressed in μg equivalent nonadecanoic acid/cm^2^. Statistically significant differences between waxy and russeted “Cox Orange Pippin” skins were evaluated using a Mann-Whitney test (p < 0.05). Error bars represent the standard deviation obtained from four biological replicates*.

A significant number of identified and unidentified pentacyclic triterpenes were found in the free lipid extract of “Cox Orange Pippin.” As expected, ursolic and oleanolic acids represented 45 and 27% of the total triterpene content in the waxy skin, respectively (Table 2). A significant decrease of these two metabolites was observed in the russeted skin, oleanolic and ursolic acid showed a 10- and 7.8-fold decrease compared to the waxy tissues, respectively. β-amyrin, which is further converted into oleanolic acid by the multifunctional CYP716A145 (André et al., 2016), was present, to a larger extent, in the waxy tissues with a fold change equal to 4.2. Interestingly, lupeol, which is also converted to betulinic acid by CYP716A145, slightly increased (*FC* = 2.4) in the russeted tissues. Surprisingly, betulinic acid, which has been previously shown to accumulate specifically in the russeted apple skin (André et al., 2012), was not detected in this fraction. Finally, germanicol was also identified from the NIST database, but no significant difference in concentration was observed between skin tissues (Table 2). According to their mass spectra and retention time, other metabolites were suspected to be part of pentacyclic triterpene family. Although they showed lower concentrations compared to the triterpenes discussed above, some of them also presented decreased concentrations in the russeted tissues.

Other metabolites from different classes were also identified (Table 2). As an example, glycerol displayed a strong decreased concentration in the russeted skin of “Cox Orange Pippin” (*FC* = 3.8). Two MAGs with C16 and C18:1 aliphatic chains were detected in the free lipid extract of both tissues (Li et al., 2007). Only the C18:1-MAG displayed a significant difference as it was only detected in the russeted tissue. Finally, the β-sitosterol was also more abundant in the waxy skin of “Cox Orange Pippin.” β-sitosterol has previously been reported in the apple peel of the “Florina” variety, but its role in the cuticle remains unclear (Verardo et al., 2003).

A large majority of the well-known cuticular wax component identified in this work showed a drastic decrease in the russeted patches of the “Cox Orange Pippin.” However, one metabolite class, namely the alkyl-hydroxicinnamates showed an inversed trend. These compounds were not detectable in the waxy skin whereas they accumulated in the suberized tissues (Table 2). Alkyl-hydroxycinnamates are synthesized by enzymes belonging to the BAHD acyltransferase family and composed by hydroxycinnamic acids conjugated with primary alcohols (Molina and Kosma, 2015; Delude et al., 2016). In the present study, a majority of alkyl-hydroxycinnamates were composed by coumarate esters followed by ferulate and caffeate esters. Primary alcohols were mostly represented by C22 and C24 chain, except for the C26-coumarate ester.

### Analysis of the depolymerized fraction

First, some alkane traces were found in the depolymerized extract (Table 3) with a predominance of the C29 alkane which was more abundant in the waxy skin (*FC* = 3.1). Other *n*-alkanes with longer chains were identified but these did not show any significant differences between the two tissues. Triterpenes were also found in the depolymerized fraction. Occurrence of alkanes and triterpenes in this fraction suggests that either some wax components stiff remained after the wax removal or that they might have been captured during the polymer assembly (intracuticular waxes). Interestingly, the distribution of the different members of this class is similar to that in the free lipid extract. Ursolic and oleanolic acids are overrepresented in the waxy tissues, whereas a strong decrease is observed in the russeted skin. Moreover, a slight accumulation of betulinic acid was also observed (Table 3). Other unidentified triterpenes, which were more abundant in the waxy tissues, were found.

**Table 3:** Distribution of the identified fatty acids (as methyl esters), fatty alcohols (as trimethylsilyl derivatives), fatty aldehydes, alkanes, triterpenes (as trimethylsilyl derivatives), hydroxycinnamic acids (as trimethylsilylated methyl esters), α,ω-dicarboxylic acids (as methyl esters), ω-hydroxyacids (as trimethylsilylated methyl esters) found in the BF3/MeOH fraction of the waxy (non-russeted) and russeted skin.

	**Non-russeted skin**		**Russeted skin**		**Mann-whitney test, *p* < 0.05**
	**Mean (μg/cm^2^)**	**Standard dev**.	**Mean (μg/cm^2^)**	**Standard dev**.	
**CARBOXYLIC ACIDS**					
C16	2.494	0.418	1.537	0.303	Sig.
C18	0.987	0.477	0.588	0.132	
C18:1	0.758	0.166	0.250	0.074	Sig.
C18:2	0.121	0.021	0.028	0.014	Sig.
C20	0.675	0.170	2.006	0.456	Sig.
C22	1.294	0.363	9.204	1.119	Sig.
C24	0.893	0.260	4.623	0.873	Sig.
C26	0.383	0.140	0.443	0.104	
C28	0.601	0.265	0.170	0.065	Sig.
C30	0.756	0.286	0.229	0.090	Sig.
**PIMARY ALCOHOLS**					
C18	0.458	0.148	0.442	0.039	
C20	0.174	0.064	0.256	0.057	
C22	0.378	0.102	2.732	0.285	Sig.
C24	1.988	0.363	3.519	0.391	Sig.
C26	0.799	0.291	0.893	0.173	
C28	0.866	0.293	0.277	0.117	Sig.
C30	0.223	0.071	0.098	0.025	Sig.
**ALKANES**					
C29	2.036	1.014	0.664	0.140	Sig.
C30	0.443	0.581	0.231	0.009	
C31	0.299	0.394	0.164	0.008	
C32	0.162	0.210	0.086	0.013	
**DICARBOXYLIC ACIDS**					
C16	0.859	0.124	2.093	0.408	Sig.
C18	0.683	0.070	2.120	0.255	Sig.
C18:1	2.174	0.162	1.614	0.288	Sig.
C18:2	0.120	0.028	0.024	0.012	Sig.
C20	0.087	0.019	0.437	0.129	Sig.
C22	0.081	0.021	0.404	0.115	Sig.
C24	0.000	0.000	0.039	0.016	
9,10-dihydroxy-C16	0.285	0.070	0.192	0.086	
9,10 dihydroxy-C18	0.707	0.061	4.627	0.876	Sig.
**HYDROXYACIDS**					
C16	2.423	0.227	1.642	0.290	Sig.
C18	10.196	0.967	1.620	2.556	Sig.
C18:1	2.221	0.193	3.999	0.467	Sig.
C18:2	3.918	0.326	1.206	0.433	Sig.
C20	0.000	0.000	0.605	0.095	Sig.
C22	0.279	0.093	2.364	0.386	Sig.
C24	0.213	0.060	0.506	0.091	Sig.
10,16-dihydroxy-C16	10.276	0.950	4.760	0.874	Sig.
9,10,18 trihydroxy-C18	6.398	0.970	7.122	0.766	
9,10,18-trihydroxy-C18:1	12.562	1.404	3.445	1.015	Sig.
HA1	1.011	0.137	0.231	0.170	Sig.
HA2	0.528	0.073	0.138	0.047	Sig.
HA3	0.554	0.076	0.592	0.120	
HA4	1.165	0.169	1.146	0.450	
**TRITERPENES**					
Lupeol	0.015	0.022	0.040	0.015	
Oleanolic acid	0.835	0.141	0.076	0.042	Sig.
Betulinic acid	0.000	0.000	0.119	0.041	Sig.
Ursolic acid	3.977	0.508	0.335	0.211	Sig.
TTP7	0.093	0.012	0.019	0.015	Sig.
TTP8	0.236	0.022	0.044	0.039	Sig.
TTP9	0.000	0.000	0.059	0.027	Sig.
TTP10	0.070	0.018	0.009	0.008	Sig.
TTP11	0.093	0.026	0.000	0.000	Sig.
TTP12	0.158	0.026	0.021	0.016	Sig.
**HYDROXYCINNAMIC ACIDS**					
*p*-Coumaric acid	1.061	0.105	0.522	0.115	Sig.
Ferulic acid	0.174	0.020	1.691	0.336	Sig.

*Quantities are expressed in μg equivalent nonadecanoic acid/cm^2^. Statistically significant differences between waxy and russeted “Cox Orange Pippin” skins were evaluated using a Mann-Whitney test (p < 0.05). Error bars represent the standard deviation obtained from four biological replicates*.

The fatty acid analysis (as methyl esters) showed significant differences in concentrations between the russeted and waxy skin (Table 3). The C16, C18:1, C18:2, C28, and C30 fatty acids were slightly, but significantly more abundant in the waxy skin. As an example, the C16, C18:1, and C18:2 fatty acids accumulated 1.6, 3.1, and 4.3 times more in the waxy tissues, respectively (Table 3). However, the most abundant fatty acids were found in the russeted tissues, which strongly accumulated C20, C22, and C24 fatty acids, with respective 2.96-, 7.1-, and 5.2-fold increase. The analysis of the depolymerized extracts also showed a significant increase of C22 and C24 chain length trimetylsilylated primary alcohols in the russeted skin with 7.7- and 1.8- fold increase, respectively (Table 3). Conversely, the C28 and C30 chain primary alcohols were more abundant in the waxy skin with 3.1- and 2.3- fold increase compared to russeted skin. As transesterification of the conjugated primary alcohols results in a free hydroxyl group, these data reflect a merged quantification of the free and conjugated primary alcohols i.e., alkyl-hydroxycinnamates. However, the differential accumulation of, C22 and C24 primary alcohols on one hand and C28 and C30 ones, on the other hand, suggests that these two groups might have been synthesized for a different purpose. Interestingly, significant amounts of *p*-coumaroyl and feruloyl-methyl esters were found in the depolymerized fraction and showed an inverse accumulation pattern between waxy and russeted tissues. The waxy skin presented a higher amount of *p*-coumaroyl-methyl ester and a lower amount of feruloyl-methyl ester compared to the russeted skin. It is noteworthy that the feruloyl methyl ester exhibited the largest difference between both tissue types (*FC* = 9.7), which might be explained by the crucial role of ferulic acid in the suberin structure and its associated waxes (i.e., alkyl-hydroxycinnamtes) (Bernards, 2002; Graça, 2015; Molina and Kosma, 2015).

The α,ω-dicarboxylic acids are major components of cutin and suberin. These precursors are esterified with glycerols on both carboxyl groups resulting in a reticular structure forming the core backbone of these polymers (Bernards, 2002; Graça, 2015). The analysis of BF_3_/MeOH depolymerized extract showed that α,ω-dicarboxylic acids concentrations (as di-methyl esters) were higher in the suberized (russeted) tissues (Table 3). This observation is in agreement with a previous work performed on *A. thaliana*, which also showed higher proportions of these diacids in suberin compared to cutin (Franke et al., 2005). As an example, in russeted tissues, C16 and C18 α,ω-dicarboxylic acids increased by 2.4 and 3.1 fold compared to the waxy tissues, respectively. The 9,10 dihydroxy-α,ω-dicarboxylic acid showed the strongest increase among this metabolite family (*FC* = 6.5). Finally, three saturated very long chain α,ω-dicarboxylic acids comprising 20, 22, and 24 carbon chains also showed, to a lower extent, a significant increase in the russeted skin (*FC* = 5 for the C20 and C22, presence for the C24). On the contrary, the C18:1 and C18:2 α,ω-dicarboxylic acid-dimethyl esters slightly decreased in the russeted tissues.

Finally, for ω-hydroxyacids, the waxy skin of “Cox Orange Pippin” showed higher concentrations in 16-hydroxydecanoic acid; 18-hydroxydecanoic acid, 18-hydroxy-9,12-octadecenoic acid, 10,16-dihydroxyhexadecanoic acid and 9,10,18-trihydroxy-12-octadecenoic acid (as trimethylsilylated methyl esters, Table 3). The 18-hydroxydecanoic acid, 18-hydroxy-9,12-octadecenoic acid and the 9,10,18-trihydroxy-12-octadecenoic acid showed remarkable differences in concentration with FC equal to 6.3, 3.2, and 3.6, respectively (*p* < 0,05). In addition, some putative ω-hydroxyacids with mid-chain modifications also displayed a slight increase in the waxy skin. Conversely, suberized skin presented a higher amount of 18-hydrodecanoic acid and, as observed for the previous classes, some members with longer chain, including the 20-hydroxyeicosanoic acid, 22-hydroxydocosanoic acid and 24-hydroxytetracosanoic acid, the highest increased being observed for the 22-hydroxydocosanoic acid (*FC* = 2.4).

### Differential expression of cutin, suberin, wax, and triterpene biosynthetic genes between russeted and waxy apple tissues

**Figure 2:**
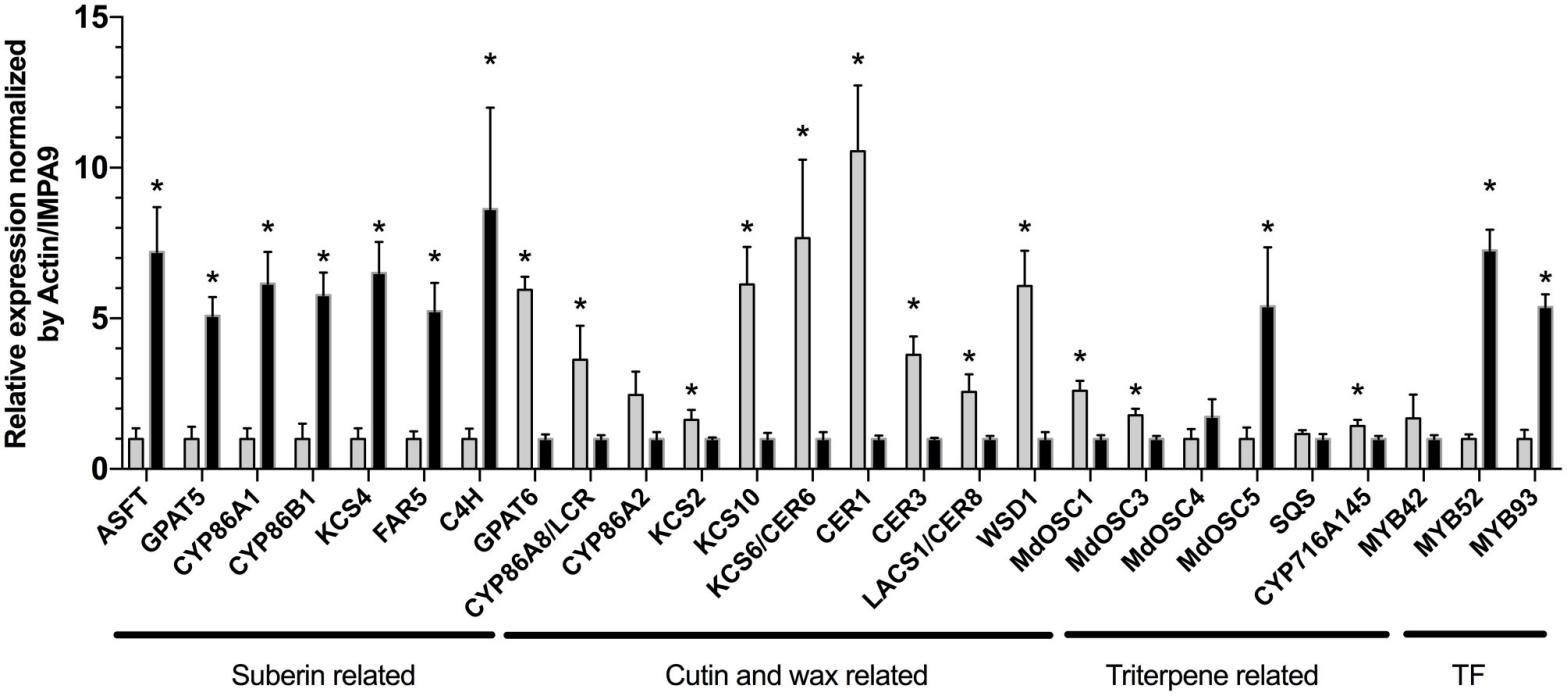
Normalized relative expression (NRq) of lipid surface biosynthesis genes performed in the waxy skin (in gray) and russeted skin (in dark). Relative quantities (Rq) are normalized by Md_Actin and Md_IMPA9 (showed as control (not normalized), NF: normalization factor). ^*^Asterisks highlight genes displaying a statistically significant difference between waxy and russeted “Cox Orange Pippin” skin obtained from a Mann-Whitney test in qbase+ v3 (*p* < 0.05). Normalized relative quantities were rescaled to the less expressed group (waxy or russet skin) to better visualize fold changes. Error bars display the standard deviation obtained from four biological replicates for each groups. ASFT, Arabidopsis suberin feruloyl transferase-like; GPAT, Glycerol-3-phosphate acyl transferase; CYP, cytochrome P450; “CYP86A1,” fatty acid hydroxylase; “CYP86B1,” very long chain fatty acid hydroxylase; FAR, fatty acid reductase; C4H, cinnamate-4-hydroxylase; CYP86A2, putative fatty acid hydroxylase, CYP86A8/LCR; CER, ECERIFERUM; LACS, long chain acyl-CoA synthetase; KCS, 3-ketoacyl-CoA synthase; WSD1, bifunctional wax ester synthase/diacylglycerol acyltransferase; SQS, Squalene synthase; OSC, oxidosqualene cyclase; CYP716A145, pentacyclic triterpene oxidase; TF, transcription factors; MYB, MYB transcription factor.

In order to support the results obtained from the free lipid and BF_3_/MeOH extracts, the expression of the apple orthologs of *A. thaliana* genes involved in the cutin, suberin, wax and triterpene biosynthesis, was also investigated in the semi-russeted “Cox Orange Pippin” variety (Figure 2; Brendolise et al., 2011; Legay et al., 2015; André et al., 2016). As observed in our previous work, the data displayed drastic changes in the expression of genes involved in these pathways between the russeted and waxy skin of “Cox Orange Pippin.”

Cutin biosynthesis genes globally showed a decreased expression in the russeted tissues. First, a gene coding for a long chain acyl-CoA synthetase 1 (*LACS1/CER8*), which is responsible for the CoA activation of the fatty acid precursors, was downregulated (Lü et al., 2009). In *A. thaliana*, C16, C18 and C18:1 acyl-CoA precursors are further hydroxylated into ω-hydroxyacids by fatty acid hydroxylases, such as the CYP86A2 or/and CYP86A8 (LACERTA, LCR) (Wellesen et al., 2001; Voisin et al., 2009). In the present study, a significant decreased expression (*FC* = 3.6) of the *LCR* gene was observed in the russeted skin, suggesting that synthesis of the cutin monomer precursors was impaired in these tissues. This decreased cutin biosynthesis was also supported by the lower expression of the *GPAT6* gene (*FC* = 5.9). In *Solanum lycopersicum*, the GPAT6 has a strong specificity for C16 and C18 carbon chain and plays a crucial role in the formation of ω-hydroxyacyl- and α,ω-dicarboxyl-glycerol monomers, which are further used as cutin building blocks (Petit et al., 2016). The expression of genes belonging to wax biosynthesis seemed to be altered in the russeted area of the “Cox Orange Pippin” skin. Three 3-ketoacyl-CoA synthase genes (*KCS2, 6* and *10*), responsible for the conversion of fatty acyl-CoA into very long chain acyl-CoA, which are further used for the wax biosynthesis, also showed a decreased expression (Millar et al., 1999; Voisin et al., 2009; Yeats and Rose, 2013). The Eceriferum *CER1* and *CER3* genes, which are also involved in the wax biosynthesis presented a similar expression pattern with fold changes equal to 10 and 3.8, respectively. Finally, the bifunctional wax ester synthase/diacylglycerol acyltransferase (*WSD1*) gene found in our previous bulk transcriptomic analysis was also tested and showed a similar trend (*FC* = 6.1). On the other hand, the expression of suberin biosynthetic genes seemed to be enhanced in the russeted skin. A *KCS4* gene displayed an increased expression in the russeted tissues (*FC* = 6.5). In *A. thaliana*, the KCS2/DAISY and KCS20 has a redundant function in cuticle waxes and root suberin biosynthesis (Lee et al., 2009). A *KCS2* gene was also induced in the russeted varieties, in our previous work (Legay et al., 2015), but the involvement of the KCS4 in the suberization process is still unclear. In *A. thaliana*, a KCS4 enzyme expressed in flowers and old siliques was unable to use the C16 and C18:1 fatty acid precursor as substrate, suggesting that this enzyme might process acyl-CoAs with longer carbon chains (Blacklock and Jaworski, 2006; Joubès et al., 2008). The expression of the *FAR5*, which encodes fatty acyl-CoA reductase and produce primary alcohols, was also significantly induced with fold change equal to 5.2. Genes for well-known CYP86A1 fatty acid hydroxylase and CYP86B1 VLCFA hydroxylase significantly increased expression in the russeted tissues (Höfer et al., 2008; Compagnon et al., 2009). Gene for GPAT5, which is responsible for the suberin specific biosynthesis of ω-hydroxyacyl- and α,ω-dicarboxyl-glycerols showed a similar expression pattern (*FC* = 5.1). The expression of *MYB52* and *MYB93* transcription factors was also induced in the russeted tissues, with *FC* = 7.2 and 5.4, respectively. In apple, MYB93 has been previously showed to be a suberin master regulator, which tightly regulate the *GPAT5, CYP86A1*, and *CYP86B1* expression (Legay et al., 2015, 2016). The orthologous gene of the *A. thaliana* suberin feruloyl-CoA acyl transferase (ASFT), which is crucial for the incorporation of ferulic acid in the suberin matrix was also significantly induced (*FC* = 7.2; Molina et al., 2009; Legay et al., 2015). Finally, the cinnamate-4-hydroxylase (C4H) involved in the hydroxycinnamic acid biosynthesis, was also strongly induced in the russeted skin (*FC* = 8.3).

Genes belonging to the pentacyclic triterpene biosynthetic pathway were also investigated (Brendolise et al., 2011; André et al., 2016). The *MdOSC1*was more and *MdOSC3* genes were more expressed in the waxy skin (Brendolise et al., 2011; André et al., 2016). Inversely, the *MdOSC5* gene was significantly up-regulated in the russeted skin. A similar trend was observed for the *CYP716A145* gene (André et al., 2016) with a slight increase (43%) in the waxy skin. Genes for the squalene synthase, which is involved in the early steps of the triterpene synthesis, and MdOSC4 were not differentially expressed between these two tissues.

## Discussion

Russeting is a major concern for the apple fruit production. Since the 60's, the complex relationship occurring between cuticle deposition and russeting has been extensively reported by multiple research teams. The current model postulates that russeting occurrence is tightly linked with defects and/or lower deposition of the cuticle on the fruit surface (Sironval and Clijsters, 1962; Walter, 1966; Faust and Shear, 1972; Skene, 1982; Knoche and Grimm, 2008; Lashbrooke et al., 2015). Our previous bulk transcriptomic analysis showed that genes involved in cutin and wax biosynthesis displayed a decreased expression in russeted apple skin, with a concomitant increased expression of the suberin biosynthetic genes (Legay et al., 2015). Compared to the fully russeted varieties, which represent an extreme case of russeting, the use of the semi-russeted “Cox Orange Pippin” provides a model which is closer to the genetic and phenotypic germplasm used in the apple production. Indeed, russeting in commercial varieties usually appears as patches, like those observed in “Cox Orange Pippin,” which gradually appear during the fruit development, whereas old heirloom fully russeted apples exhibit this strong phenotype at very early stages of fruit growth (Sironval and Clijsters, 1962; Walter, 1966; Faust and Shear, 1972).

**Figure 3:**
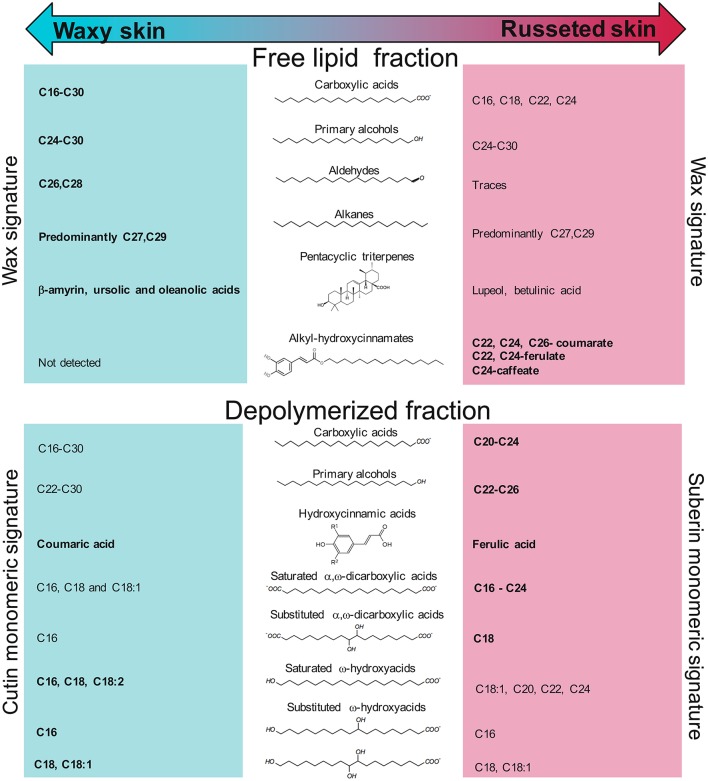
Overview of the metabolites found in the free lipid and depolymerized fractions of the “Cox orange Pippin” waxy and russeted skins. Compounds highlighted in bold indicate the global higher concentrations of their respective family in the tissue.

In the present study, the total wax concentration observed in the waxy tissue (537 μg/cm^2^, 20.5% of the cuticle sample) is in accordance with previous reports, which described total wax concentrations ranging from 366 to 1,038 μg/cm^2^ depending on the variety (Belding et al., 1998). This suggests that the non-russeted skin surface of Cox Orange Pippin might be considered as moderately rich in wax. Alternatively, russeted apple tissues showed a massive decrease in this total wax concentration, which reach only 87.7 μg/cm^2^ (3% of the cuticle sample). This supports the hypothesis that a thinner and/or damaged cuticle is a crucial factor leading to russeting in apple (Skene, 1982; Knoche and Grimm, 2008; Lashbrooke et al., 2015). This decrease was observed in nearly all components of waxes and included the very long chain primary alcohols, aldehydes, fatty acids and alkanes, with carbon chains longer than 24 carbons (Table 2, Figure 3) and suggests that a decreased very long chain fatty acid (VLCFA) biosynthesis occurs in these russeted areas. This was also supported by the decreased expression of genes coding for *KCS2, KCS6*, and *KCS10*, which are implicated in the elongation of C16 and C18 acyl-CoA into very long chain acyl-CoA, the latter being used as precursors for the different classes of metabolites comprised in these cuticle waxes (Millar et al., 1999; Lee et al., 2009; Lee and Suh, 2013). Finally, decreased concentrations in C16 and C18 fatty acid precursors were also observed suggesting that the core fatty acid biosynthesis which occurs in the plastids is also altered in the russeted tissues.

The following steps of the cuticular wax biosynthesis are still under investigation. However, it is suggested that the wax biosynthesis pathway follows two distinct branches (Millar et al., 1999; von Wettstein-Knowles, 2001). The reductive pathway produces even number carbon chain numbers as found in aldehydes and primary alcohols. Aldehydes are considered as intermediate compounds between the acyl-CoAs and primary alcohols (von Wettstein-Knowles, 2001). On the other side, the decarbonylation of fatty acyl-CoAs might be involved in the biosynthesis of classes sharing odd carbon chain number, as observed for the alkanes. These latter compounds are further hydroxylated into secondary alcohols and ketones. In the present work, it is noteworthy that the major primary alcohols and aldehydes were composed of 26- and 28-carbon chains whereas alkanes showed C27- and C29-carbon chains. Thirty-carbon chains were only observed for the free fatty acids, which strongly accumulated in the waxy skin of “Cox Orange Pippin” (Table 2, Figure 3). As VLCFAs are synthesized from very long chain acyl-CoA, the C29 *n*-alkanes biosynthesis might result from the decarbonylation of a C30 acyl-CoA common precursor. CER1, CER3 and CER8 are also involved in the alkane biosynthesis (Aarts et al., 1995; Rowland et al., 2007; Lü et al., 2009). Apple orthologous genes of *CER1* and *CER3*, which were found in our previous RNA-Seq data, were more expressed in the waxy tissues of “Cox Orange Pippin,” suggesting that they might indeed play a role in the biosynthesis of apple skin waxes. We noticed some trace of n-alkanes in the depolymerized fraction, which might (i) remained after the wax removal or (ii) have been confined in the polymer matrix, probably in the cutin, and released during the depolymerization process.

As opposed to the different wax compounds described above, the “Cox orange Pippin” russeted fruit skin specifically accumulated some alkyl-hydroxycinnamates (Table 2, Figure 3). These metabolites, which are composed of hydroxycinnamic acids conjugated with primary alcohols, have been previously associated with the suberization process in *A. thaliana* and *S. tuberosum* to name a few (Schreiber et al., 2005; Serra et al., 2010; Kosma et al., 2012, 2015; Molina and Kosma, 2015; Delude et al., 2016). In our work, a majority of coumarate conjugates have been found, followed by ferulates and caffeates. Primary alcohols esterified with these hydroxycinnamic acids are mostly represented by 22- and 24-carbon chains length (Table 2, Figure 3). This was not in accordance with the primary alcohols distribution observed in the free lipids extract, which displayed predominantly 26- and 28- carbon chains. However, primary alcohols found in the depolymerized extract showed a completely different pattern with an enhanced accumulation of C22 and C24 chains in the russeted skin (Table 3, Figure 3). In *A. thaliana* roots, the large majority of the primary alcohols found in the suberized tissues are in the form of alkyl-hydroxycinnamates (Delude et al., 2016). In apple, it might be conceivable that a similar process occurred and explains the concordant distribution of primary alcohols observed in the alkyl-hydroxycinnamates and those identified in the depolymerized extract. This suggests that the differentially expressed *KCS2 and KCS4* genes, observed in this work and in our previous transcriptomic data (Legay et al., 2015), might be responsible for the specific synthesis of these C22 and C24 acyl-CoA. These are partly converted into primary alcohols by fatty acyl CoA reductase enzymes, such as FAR5, which expression was enhanced in the present work (Domergue et al., 2010). In *A. thaliana*, the *kcs2* loss-of-function mutant displayed a significant decreased content in C22 and C24 VLCFAs (Lee et al., 2009). The suberin biosynthetic process occurring in the russeted skin might induce *KCS2* genes which produce essentially C22 and C24 chains for further synthesis of these alkyl-hydroxycinamates. Inversely, the *KCS2, KCS6*/*CER6*/*CUT1* and *KCS10* genes, which are up-regulated in the waxy skin, might produce longer carbon chain acyl-CoA, which are further used for the cutin wax production, including the C26 and C28 primary alcohols observed in the present work. As an example, the *cut1*-loss of function mutant led to an over-accumulation of C24 chain-length wax components, suggesting that KCS6 is responsible for the elongation of the C24 acyl-CoA into longer precursors (Millar et al., 1999).

Pentacyclic triterpenes are major components of the fruit skin waxes in apple (André et al., 2012). In waxy varieties, the total triterpene content can reach up to 70% of the total waxes content depending on the genetic background of the variety and the environmental factors (André et al., 2012). Three main triterpene series, namely the ursane, oleane, and lupane series, have been identified in the apple skin, so far. The distribution of the different series of triterpene seems to be tightly linked with the degree of skin russeting. Indeed, recent studies showed that the ursane and oleane series strongly accumulate in the waxy tissues, whereas the lupane series seems to be specifically synthesized in the russet skin (Brendolise et al., 2011; André et al., 2012, 2016). Ursolic and oleanolic acids concentrations strongly decreased in the russeted skin, suggesting that in addition to the fatty acyl derivatives, the alicyclic component of apple waxes is also altered in this tissue (Table 2). However, a recent study showed that the transpiration barrier efficiency is mainly driven by the fatty acyl derivatives content in the intracuticular waxes (Jetter and Riederer, 2016). This suggests that in russeted apple tissues, decreased concentrations in ursolic and oleanolic acids might not have a strong impact on the transpiration barrier efficiency, but further are needed to validate this hypothesis. This observation was also supported by the decreased expression of the *MdOSC1* and *MdOSC3* genes in the russeted skin. MdOSC1 produces mainly α-amyrin, which is further converted into ursolic acid by the CYP716A145 oxidase. *MdOSC3*, which is highly homologous to *MdOSC1*, probably because of the ancestral genome duplication of the *Pyrae* ancestor, has a similar function (Brendolise et al., 2011; André et al., 2016). Conversely, *MdOSC5*, which has been recently described as a crucial enzyme involved in the synthesis of lupeol (the betulinic acid precursor), was up-regulated in russeted tissues (André et al., 2016). Previous works showed that betulinic acid predominantly accumulates in the russeted tissues (André et al., 2012). However, betulinic acid was only detected in the depolymerized fraction. To our knowledge, triterpenes are not covalently linked to the cutin and suberin polymers (André et al., 2012, 2016). Thus, we can speculate that it might be embedded in this polymeric matrix (probably suberin). Finally, it is noteworthy that other minor unidentified pentacyclic triterpenes, which display differential accumulations, have been detected in both fractions. However, standards are currently lacking to identify them properly and it is not yet possible to identify them. Finally, the *CYP716A145* gene displays a slight increase (43%) in the waxy skin. This multifunctional enzyme is responsible for the conversion of α-amyrin, β-amyrin and lupeol into ursolic, oleanolic and betulinic acids, respectively (André et al., 2016). Altogether, these observations strengthened the present knowledge about the tight relationship between the suberization process and the pentacyclic triterpenes synthesis. As ursolic and oleanolic acids are major component of the non-russeted skin waxes, their decreased accumulation might also have an impact on the integrity of the cuticle. Thus, it would be interesting, in further works, to evaluate their role in the development of cuticle microcracking.

The boron trifluoride-methanol (BF_3_/MeOH) transesterification method is commonly used to depolymerize either cutin or suberin (Franke et al., 2005; Gou et al., 2009; Panikashvili et al., 2009). It is worth noting that, in the present experimental design, these two polymers are likely to be present in the russeted skin of the “Cox Orange Pippin” variety, whereas a large majority of cutin monomers are expected in the waxy tissue. The total amount of released monomers did not significantly changed between waxy and russeted tissues with 80.2 μg/cm^2^ (3.06% of the starting material) and 73.3 μg/cm^2^ (2.27% of the starting material), respectively. This suggests that other polymers and compounds, such as polysaccharides, cutan or lignin, which are recalcitrant to the transesterification process might remain in the skin tissue. As example, the polyaromatic domain of suberin, which is considered as a lignin-like polymer, might probably remain in this fraction (Bernards, 2002). Moreover, cutan, which is also a lipidic polymer found in the apple cuticle, might also remain in the residual fraction as its reticulation seems to be caused by ether and C-C bounds, the latter being recalcitrant to the BF3/MeOH treatment (Johnson et al., 2007; Yeats and Rose, 2013). Finally, cell wall polysaccharides, which are embedded in the cuticular layer or linked to the polyaromatic domain of suberin might also remain in this fraction.

The analysis of the depolymerized extract showed strong differences between the metabolic profiles obtained from the russeted and waxy skin of “Cox Orange Pippin.” As example, the distribution of fatty acids was particularly interesting and differed from the results obtained in the free lipid extract. Suberized tissues displayed increased concentrations of VLCFAs ranging from C20 to C24 chains, whereas a decreased accumulation of C16, C18, C18:1, and C18:2 was observed (Table 3, Figure 3). In plants, the composition of suberin-associated fatty acids seems to vary among species and tissues. In *A. thaliana* roots, the analysis of the suberin monomers revealed a majority of C20 and C22 fatty acids whereas the *S. tuberosum* suberin exibits longer carbon chains with a majority of C24–C28 fatty acids (Graça and Pereira, 2000b; Franke et al., 2005). *A. thaliana* seed coat suberin is composed of a majority of C16, C20, and C24 chains (Molina et al., 2006). Finally, the bark suberin monomeric contents of *P. menziesii* and *Q. suber* present higher proportions of the C20–C24 fatty acids as observed in our work (Graça and Pereira, 2000a). Caution should be taken with the present results because these fatty acids are probably released from both cutin and suberin. However, as observed for the alkyl-hydroxycinnamate-associated primary alcohols, our results suggest a suberin specific biosynthesis of C20–C24 (predominantly C22) very long chain acyl-CoA precursors in the russeted tissues (Lee et al., 2009; Legay et al., 2015). Interestingly, small amount of C20, C22, and C24 precursors were also observed in the waxy tissues and might be due to the presence of suberized lenticels on the whole surface of the fruit. Lenticels are small pores spread over the fruit surface, which have been first linked to high transpiration rate. However, more recent studies showed that this transpiration rate was not correlated with the size and the number of lenticels due to either suberin or wax deposition on their surface (Martin and Rose, 2014).

In *A. thaliana*, the analysis of the cutin composition indicated a quasi-exclusive occurrence of C16 and C18 monomers, suggesting that the shorter carbon chain fatty acids, found in a higher amount in the waxy apple skin, might be part of the cutin polymer and that their synthesis is impaired in the russeted tissue (Bonaventure et al., 2004). The composition of the ω-hydroxyacids is more complex but seemed to follow a similar trend compared to fatty acids. Indeed, waxy skin contained increased amount of C16, C18, C18:2 whereas a specific accumulation of C18:1, C20, C22, and C24 ω-hydroxyacids was observed in the suberized skin (Table 3). In *A. thaliana* roots, ω-hydroxyacids are mainly composed of C16, C18, C20, and C22 aliphatic chains with a strong predominance of the 18-hydroxyoctadec-9-enoic acid (C18:1 ω-hydroxyacid, Franke et al., 2005), whereas C18:1, C18:2, C22, and C24 ω-hydroxyacids are mostly represented in the *A. thaliana* seed coat suberin (Molina et al., 2006). In the potato periderm, a massive amount of C18:1 ω-hydroxyacid has been also found and, to a lower extent, some C16, C22, C24, C26, and C28 ω-hydroxyacids (Graça and Pereira, 2000b). Finally, the bark of *Q. suber* displays a majority of C22, C24 ω-hydroxyacids (Graça and Pereira, 2000a). In our study, the specific accumulation of these very long chain ω-hydroxyacids in the apple suberized skin was supported by the increased expression of the VLCFA hydroxylase (*CYP86B1*) gene (Compagnon et al., 2009). Moreover, an increased expression of *GPAT5* was observed in the russeted tissues. In *A. thaliana*, GPAT5, which synthetizes the suberin ω-hydroxyacyl- and α,ω-diacyl-glycerol building blocks, has a strong affinity for the very long chain (>C18) ω-hydroxyacids and α,ω-dicarboxylic acids (Beisson et al., 2007). Altogether, these data support the idea that C20, C22, and C24 ω-hydroxyacids might constitute specific precursors of suberin in apple. Additionnally, the C18:1 ω-hydroxyacid, which strongly accumulates in the *A. thaliana* roots (Franke et al., 2005) and the russeted potato periderm (Graça and Pereira, 2000b), is the only short chain ω-hydroxyacid displaying an increased concentration in the russeted apple skin. We might speculate that this compound might be produced by the CYP86A1 fatty acid hydroxylase (Höfer et al., 2008), which showed a significant increased expression in our RT-qPCR results. Some mid-chain hydroxylated ω-hydroxyacids showed a higher concentration in the waxy skin, suggesting that they might be part of the cutin polymer. These might be synthesized by the preliminary action of some cutin specific epoxidase, such as the *A. thaliana* CYP77A4 (Sauveplane et al., 2009), which was also more expressed in the waxy skin in a previous work (Legay et al., 2015). Epoxidized fatty acids might be further hydroxylated by some epoxide hydrolase to yield mid-chain hydroxylated fatty acids, but no candidate has been found yet (Yeats et al., 2012a).

The core structural backbone of both cutin and suberin is composed of α,ω-dicarboxylic acids esterified to glycerols. These are crucial components of the cutin and suberin building blocks despite reduced amounts were found in cutin (Moire et al., 1999; Beisson et al., 2007; Yang et al., 2010, 2016). Surprisingly, we found increased amount of free glycerol in the waxy tissues. This might be explained an enhanced activity of the CD1 (cutin synthase) polymerization enzyme, which release a glycerol moiety from each MAG condensed (Yeats et al., 2012b). Previous studies showed that suberin shows a high content in α,ω-dicarboxylic acids, as compared to cutin (Franke et al., 2005). A similar tendency was observed in apple, which showed an increased accumulation in the majority of the identified α,ω-dicarboxylic acids. Interestingly, the highest contents were observed for the shorter carbon chains α,ω-dicarboxylic acids, such as hexadecanedioic, octadecanedioic and 9,10-dihydroxyoctadecanedioic acids, whereas eiocosanedioic, docosanedioic and tetracosanedioic acids were less abundant. Again, the apple suberization process seemed to specifically produce C20, C22, and C24 α,ω-dicarboxylic acids, whereas shorter carbon chains α,ω-dicarboxylic acids were predominant in the waxy apple skin (Table 3, Figure 3). The same trend is observed in *S. tuberosum, A. thaliana* roots and *P. menziesii* (Graça and Pereira, 2000a,b; Franke et al., 2005). Only suberin monomers obtained from the *Quercus suber* bark and *A. thaliana* seed coats displayed predominant or large amount of very long chain α,ω-dicarboxylic acids (Graça and Pereira, 2000a; Molina et al., 2006). The synthesis of these α,ω-dicarboxylic acids remains unclear, some authors argue that the consecutive action of alcohol dehydrogenase and aldehyde dehydrogenase on ω-hydroxyacids precursors might support the synthesis of these crucial compounds (Kurdyukov et al., 2006; Franke and Schreiber, 2007). The second putative DCA synthesis path might involve cytochrome P450-based enzymes being able to perform both steps of this carboxylation process. As example, a CYP94A5 isolated from *Nicotiana benthamiana* and expressed in *Saccharomyces cerevisiae* is able to convert the 18-hydroxy-9,10-epoxystearic acid into its corresponding DCA (Le Bouquin et al., 2001).

Finally, coumarate and ferulate methyl esters were detected in both russeted and waxy tissues, suggesting that they were covalently linked within the cutin and/or suberin polymeric matrix, or conjugated into other compounds, such as alkyl-hydroxycinnamates or triterpene conjugates (Kosma et al., 2012; André et al., 2013; Table 3, Figure 3). Higher concentration of ferulic acid in the russeted skin is obvious as it is one of the major suberin structure signature (Bernards, 2002; Franke et al., 2005; Molina et al., 2006; Graça, 2015; Vishwanath et al., 2015). Hydroxycinnamic acids are thought to be part of the suberin polyphenolic domain of suberin which is anchored in the cell wall (Bernards et al., 1995; Bernards, 2002). Moreover, ASFT is responsible for the linkage of ferulic acid with ω-hydroxyacids resulting in feruloyl-acyl esters, which are considered as crucial suberin building blocks (Molina et al., 2009). An apple orthologous gene of *ASFT*, which has been found in our previous transcriptomic profiling of the russet trait, displayed a strong increased expression in the russeted tissues of “Cox Orange Pippin” (Legay et al., 2015). Covalently linked coumaric acid, possibly to cutin, has been already described in apple, but not ferulic acid (Riley and Kolattukudy, 1975); this might be explained by the fact that this study did not comprise russeted varieties. Conversely, a fairly detectable amount of coumaric acid was found in the russeted skin and even more in the waxy skin, suggesting that coumaric acid might be more related to the cuticle development but its exact involvement in this matrix remains unclear (Riley and Kolattukudy, 1975).

As a whole, the present data depict for the first time the complex structure of the waxy and russeted apple skins. Our previous transcriptome profiling study showed an impaired expression of genes involved in the cuticle deposition in the russeted varieties with a concomitant increased expression of genes involved in the suberin biosynthetic pathway. Recent studies postulated that an efficient cuticle is crucial to prevent any occurrence of suberization on the fruit surface (Knoche and Grimm, 2008; Khanal et al., 2012; Lashbrooke et al., 2015). Our data clearly support this hypothesis. Indeed, in the russeted skin patches of the “Cox Orange Pippin,” a massive decrease of common wax components was observed. These included primary alcohols, alkanes, fatty acids and aldehydes, along with the pentacyclic triterpenes ursolic and oleanolic acids. This decrease in the wax content is also accompanied by a reduced synthesis of cutin, which is composed by a majority of 16- and 18-carbon chain ω-hydroxyacids and α,ω-dicarboxylic acids. The concomitant decreased concentrations of cutin and wax components, together with the expression pattern of their biosynthetic genes, suggest that a common regulatory network might regulate these pathways. It would be interesting to investigate whether some cuticle deposition master regulators, such as *MdSHN3* and/or a *AtMYB94* apple orthologous genes (Lashbrooke et al., 2015; Lee and Suh, 2015), are able to alter the triterpenic acid contents in the apple fruit skin.

Suberin, which accumulates in russeted patches, was similar to the previously reported composition investigated in other species (Graça and Pereira, 2000a,b; Franke et al., 2005; Molina et al., 2006). In addition to the characteristic ferulic acid, C20, C22, and C24 very long chain backbone precursors, which are further used for the synthesis of primary alcohols, fatty acids, ω-hydroxyacids, α,ω-dicarboxylic and possibly the suberin specific non-covalently linked alkyl-hydroxycinnamates, constituted the main characteristic of the apple suberin. The tight interplay between the decreased cuticle synthesis and suberin deposition is evident and the central remaining question now relates to the causes leading part of the fruit epidermis to a decreased cuticle deposition. Further studies have therefore to be performed to answer this crucial question.

## Author contributions

SL, GG, CA, and JH designed the experimental setup. SL, EC, and GG performed the sampling. SL and GG performed the transriptomic work (RNA extraction, QC control, RT-qPCR). SL, EC, CA, and CG performed the analytical chemistry work, the GC-MS data extraction, metabolite identification. SL and GG performed the statistical analysis. SL, GG, and EC wrote the article. SL, EC, CA, CG, JH, and GG involved in the manuscript refinement.

### Conflict of interest statement

The authors declare that the research was conducted in the absence of any commercial or financial relationships that could be construed as a potential conflict of interest.
